# Revolution of Biotechnology with CRISPR

**DOI:** 10.1038/s12276-025-01452-x

**Published:** 2025-07-31

**Authors:** Kyungjae Myung

**Affiliations:** 1 Center for Genomic Integrity, Institute for Basic Science, Ulsan, Republic of Korea; 2 Department of Biomedical Engineering, Ulsan National Institute of Science and Technology, Ulsan, Republic of Korea

**Keywords:** Genetic engineering, Mutagenesis

CRISPR–Cas9 is a groundbreaking genome-editing tool that has transformed biomedical research. Originally derived from a bacterial defense system, CRISPR (clustered regularly interspaced short palindromic repeats) functions as an adaptive immune mechanism that protects bacteria from viral infections^1^. This system relies on short RNA sequences from past viral encounters to guide the Cas9 nuclease to specific DNA targets, allowing precise cleavage of foreign genetic material. Scientists have harnessed this mechanism to develop a powerful and versatile tool for targeted genome editing in various organisms, including humans, plants and model organisms used in biomedical research^2^.

The CRISPR–Cas9 system consists of two key components: a guide RNA (gRNA) and the Cas9 protein^1^. The gRNA is designed to complement a specific DNA sequence, directing Cas9 to the target site. Upon binding, Cas9 induces a double-strand break (DSB) at the designated location. The cell then repairs the break through one of two major pathways: nonhomologous end joining (NHEJ), which often results in small insertions or deletions (indels) that can disrupt gene function, or homology-directed repair, which allows precise genetic modifications using a donor DNA template^3^. These capabilities make CRISPR–Cas9 invaluable for gene knockout studies, correction of disease-causing mutations and the development of gene therapies.

Compared with earlier genome-editing technologies such as zinc finger nucleases and transcription activator-like effector nucleases, CRISPR–Cas9 offers several advantages, including ease of design, cost-effectiveness and high specificity^4^. Researchers can rapidly generate gRNAs targeting virtually any genomic sequence, making CRISPR–Cas9 an accessible and powerful tool. Ongoing improvements, such as high-fidelity Cas9 variants, base-editing and prime-editing systems, continue to enhance the precision and efficiency of genome editing, reducing the risk of unintended off-target effects^5^.

## Advances in CRISPR–Cas9 research

This issue highlights several cutting-edge developments in CRISPR–Cas9 research.

Ruis et al. explore the molecular repair mechanisms of DNA breaks induced by CRISPR–Cas9. In addition to the well-characterized homology-directed repair and nonhomologous end joining pathways, they introduce a novel repair mechanism known as CRISPR–homology-mediated end joining (HMEJ), which probably operates through a single-strand annealing process ^6,7^. Given its efficiency in gene targeting, researchers are investigating HMEJ-mediated repair for potential gene therapy applications.

Lee et al. provide an overview of recent advancements in prime editing, a technique that introduces point mutations, insertions or deletions without generating DSBs^8^. Prime editing uses a Cas9 nickase fused to a reverse transcriptase (MMLV-RT) and guided by a prime-editing gRNA, which contains both a spacer sequence for target recognition and a reverse transcriptase template. Unlike base editors, which are restricted to specific nucleotide conversions (for example, C-to-T and A-to-G) and may induce off-target mutations due to deaminase activity, prime editors enable nearly any base substitution with greater precision and fewer unintended effects. This flexibility makes prime editing a superior option for precise genome modifications.

Kim et al. review the integration of artificial intelligence (AI) with CRISPR–Cas9 technology. Their article discusses AI-driven approaches for gRNA design, off-target prediction, optimization of editing efficiency and the development of novel CRISPR systems. With rapid advances in AI, CRISPR–Cas9 technology is expected to achieve even greater precision, efficiency and safety in the near future.

## CRISPR–Cas9 in biomedical research

CRISPR–Cas9 has been widely used to study fundamental biological processes, including DNA metabolism, development, physiology and pathology. Cheng et al. review how CRISPR–Cas9 has been utilized as a genome modification tool to introduce knockouts, structural variations and epigenome edits, particularly in a multiplexed manner. In addition to the wild-type Cas9 protein, transcriptional control of genes has been achieved using CRISPR activation and CRISPR interference, which use a nuclease-dead dCas9 enzyme fused with either a transcriptional activator or repressor ^9,10^. The review also highlights a recent innovation in CRISPR-based cancer therapy, known as cancer-specific INDEL attacker (CINDELA), which selectively induces DSBs at indel mutations in tumor genomes^11^.

Varshney et al. discuss advances in CRISPR–Cas9 technology for high-throughput mutagenesis, knock-in strategies and large-scale genetic screens in vertebrate organisms. Their review examines how CRISPR–Cas9 has been used in functional genomics research and disease modeling. Furthermore, they introduce efforts to modify higher-order chromatin organization, particularly through the manipulation of topologically associated domains.

CRISPR–Cas9 has also been used to visualize specific genomic loci. Early approaches were limited to detecting repetitive sequences owing to technical constraints. Kwon et al. review innovative strategies developed to overcome these limitations, including signal amplification techniques, background noise reduction and improved genomic resolution. Lastly, Park et al. discuss potential adverse effects of CRISPR–Cas9-based genome labeling, citing reports that CRISPR–dCas9 binding to DNA can cause replication blockage, DNA–RNA hybrid triplex (R-loop) formation, subnuclear localization changes and unintended alterations in gene expression.

## Future perspectives

This issue presents a series of review articles that illustrate the diverse applications of CRISPR–Cas9 in biomedical research. As this field continues to evolve, we are witnessing the early stages of a technological revolution with the potential for groundbreaking discoveries. Beyond basic biomedical research, CRISPR–Cas9 has far-reaching applications in medicine, agriculture and biotechnology^2^. These applications, extensively reviewed elsewhere, include the treatment of genetic disorders, the development of disease-resistant crops and the engineering of microbial strains for industrial use. However, ethical and safety concerns remain, particularly regarding germline editing and unintended genetic consequences^12^. As researchers continue to refine CRISPR–Cas9 technology, addressing these challenges will be crucial in ensuring its safe and effective application in diverse fields.
